# Tropical Veterinary Medicine

**Published:** 1903-09-12

**Authors:** 


### The Hospital.

A JOURNAL OF

The Medical Sciences and Hospital Administration.

VOL. XXXIV.—NO. 885. SEPTEMBER 12, 1903.

### TROPICAL VETERINARY MEDICINE.

THE establishment, in connection with the Liverpool University, of a school for the study and teaching of the diseases incidental to animals in the tropics is a step the wisdom of which can hardly be too highly estimated. We are manifestly within sight of a period at which the work expended upon the analogous diseases of the human subject may be expected to bear fruit in the direction of opening up some of the fairest portions of the earth's surface to the industry and enterprise of races which have hitherto been excluded from them, and this industry and enterprise have in the past been nearly as much crippled by the impossibility of employing beasts of burden as by the liability of man himself to perish under maladies peculiar to certain conditions of temperature or of locality. There seems to be every prospect that, in this and other European countries, the horse may soon come to be numbered among the superfluities of the wealthy, and that the real business of the roads will be accomplished by some form of automotor; but as yet roads have not come into existence in many places into which it is eminently desirable that man should penetrate. Even when the motor is completely established in this country, so that the innocent and lamb like driver thereof will be able to discontinue his present bleatings anent the oppression exercised by the police wolf, horses and oxen will still be imperatively required as essential to the advance of civilisation in the tropics, and both animals are liable to perish there from strange diseases, usually of excessive virulence and apparently of extreme infectiveness. The idea of the extension of the teaching of the Liverpool School of Tropical Medicine so that it should embrace the diseases of animals as well as those of man, appears to have been first suggested by the late Professor Nocard of Paris, who, in an address which he gave at Liverpool, illustrated the requirements of the case by an account of an epizootic malady which worked great havoc in the Mauritius. It appears that the sugar planters of the island were accustomed to obtain from Madagascar the horses necessary for the cultivation of their land and for the carriage of their crops; but the demands of the British Government, arising from the war in South Africa, so much increased the prices in Madagascar as to compel the planters to seek another source of supply. This was obtained in India, and a shipload of Indian horses was dispatched to the Mauritius. On the voyage many of the horses sickened and some died from a malady which nobody on board understood, but the survivors, when landed in the island, soon recovered, and were sold and dispersed abroad. Wherever they went they communicated disease to other horses which were brought into proximity with them and a veritable equine pestilence was the result—a pestilence which almost deprived the island of horses and which brought the planters to the brink of ruin. In course of time skilled investigation was brought to bear upon the subject, and it was discovered that the original disease had been occasioned by a blood parasite which, presumably, was taken from the imported horses by some of the blood sucking flies of the island and by them communicated to the horses previously there. The apparently recovered imported horses seem to have played much the same part that is played by negro children in the tropics—that of seemingly healthy sources of supply of a parasite which is destructive to the unacclimatised. The parasite, in this case, was not the trypanosoma of the tse-tse fly, but appears to have been of an allied species. Professor Nocard pointed out that an examination of the blood of the convalescent imported horses would in all probability have detected the parasite, and would have prevented the whole of the mischief which ensued from their introduction. His words did not fall upon deaf ears, but induced the authorities of the Liverpool University to establish a department of comparative pathology, and to throw it open for the instruction of veterinary students and practitioners, who can now be received, and for whom apartments and accommodation have been provided. To have gone through a course of study there will, no doubt, soon be regarded as a necessary part of the qualifications of every veterinary practitioner who desires to seek his fortune in the Colonies.

It was eminently fitting that the suggestion which led to the establishment of the new department should proceed from a French savant, for comparative pathology was studied in France long before its value and importance were at all realised in this country. We remember a review of Rayer's "Comparative Medicine," which appeared in an English medical journal 40 years ago, and in which the illustrious physician to the Emperor was ridiculed for his attempt to find, in the diseases of calves and jackasses something which would throw light upon those of his countrymen. We have now happily passed beyond the phase of thought which these words would indicate, and have come to recognise that the essential relationship of our bodily frame to that of the lower animals does indeed imply a frequent similarity between the diseases to which they and ourselves are respectively prone. The instruction given at Liverpool can hardly fail to obviate some of the difficulties which now impede the full employment of animals for the purposes of carriage and draught in many tropical countries, and at the same time it may render important help in elucidating the nature and causes of diseases which derive their chief importance from their incidence upon mankind.

